# Ab Initio Potential Energy Surface for NaCl–H_2_ with Correct Long-Range Behavior

**DOI:** 10.1021/acs.jpca.3c07687

**Published:** 2024-01-25

**Authors:** Priyanka Pandey, Chen Qu, Apurba Nandi, Qi Yu, Paul L. Houston, Riccardo Conte, Joel M. Bowman

**Affiliations:** † Department of Chemistry and Cherry L. Emerson Center for Scientific Computation, Emory University, Atlanta, Georgia, 30322, United States; ‡ Independent Researcher, Toronto ON M9B 0E3, Canada; § Department of Physics and Materials Science, University of Luxembourg, Luxembourg City L-1511, Luxembourg; ∥ Department of Chemistry and Chemical Biology, Cornell University, Ithaca, New York 14853, United States; ⊥ Department of Chemistry and Biochemistry, Georgia Institute of Technology, Atlanta, Georgia 30332, United States; # Dipartimento di Chimica, Università Degli Studi di Milano, Via Golgi 19, Milano 20133, Italy

## Abstract

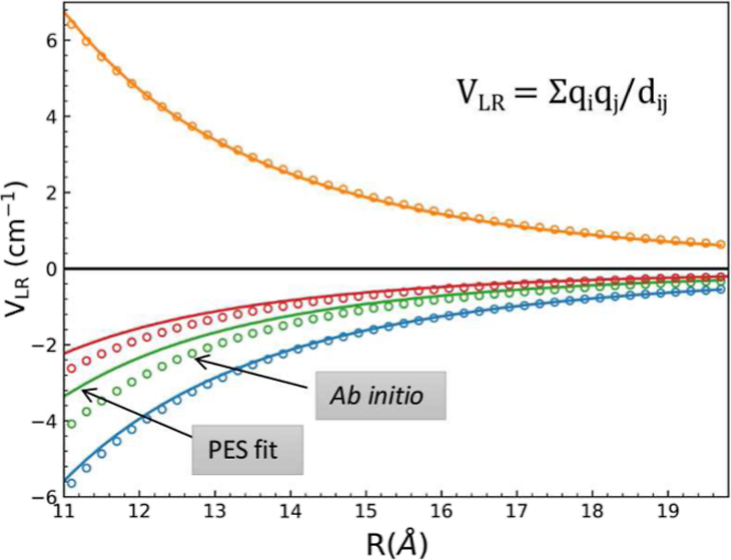

We report a full dimensional ab initio potential energy surface for NaCl–H_2_ based on precise fitting of a large data set of CCSD(T)/aug-cc-pVTZ energies. A major goal of this fit is to describe the very long-range interaction accurately. This is done in this instance via the dipole–quadrupole interaction. The NaCl dipole and the H_2_ quadrupole are available through previous works over a large range of internuclear distances. We use these to obtain exact effect charges on each atom. Diffusion Monte Carlo calculations are done for the ground vibrational state using the new potential.

## 1 Introduction

Molecular hydrogen is the fundamental component in the interstellar medium and is prevalent in most interstellar environments. Collisions involving molecular hydrogen interacting with itself and other rotationally and vibrationally excited molecules are the essential components of astrophysics to understand the evolution of planetary gases.^1,2^ Since replicating the precise conditions of astrophysical environments in the laboratory is challenging, most collision studies involving molecular hydrogen and other molecules rely on quantum scattering calculations, necessitating an accurate understanding of the relevant interaction potential energy surface (PES). In recent years, researchers have addressed the potential energy surface of various systems involving molecular hydrogen, including H_2_ + H_2_,^3^ SiO_2_–H_2_,^4^ CN–H_2_,^5^ CO–H_2_,^6^ and others.^7,8^ Apart from these predominant molecules, the formation of molecules composed of elements with lower abundances also occurs. For instance, recently, sodium chloride has been discovered in Europa,^9^ in the disk around Orion Source I,^10^ as well as in other locations.^11,12^ Hence, the computation of a full-dimensional PES for the NaCl–H_2_ system is required to understand the collisions between them.

Previously, experimental and theoretical studies have been conducted to investigate the behavior of H_2_ on crystalline solid surfaces and in aqueous solutions of NaCl. For instance, Ewing, Heidberg, and others conducted multiple experiments to explore the adsorption of H_2_ on annealed NaCl films and NaCl(110) using infrared (IR) spectroscopy^13−16^ and He atom scattering.^17^ Additionally, Monte Carlo simulations and perturbation theory calculations were utilized to investigate the structure of monolayer and bilayer films of H_2_ molecules adsorbed on NaCl(001) at different temperatures.^18^ Recently, Zhu et al. developed an accurate model of H_2_ solubility in aqueous NaCl solution.^19^ To the best of our knowledge, no NaCl–H_2_ PES exists addressing the interaction between NaCl and H_2_.

The accuracy of the PES for many atom systems, including biomolecules, condensed matter, and macromolecules, depends on the precise depiction of both short-range and long-range interactions. Therefore, accurately describing the long-range interaction potential is crucial to PES calculation^20^ and understanding phenomena such as molecular collisions,^21,22^ dispersion,^23^ and extended charge transfer.^24,25^ To achieve this goal, modeling long-range interactions using electrostatic, induction, and dispersion components yields computationally efficient and precise results.^26^

Describing long-range interactions is a challenge for the large class of atom-centered machine learning methods that use a strict cutoff for the interaction range to represent potentials.^27,28^ Quoting from Behler and co-workers in 2021, “Machine learning potentials···[that] rely on local properties···are unable to take global changes in the electronic structure into account, which result from long-range charge transfer or different charge states.”^29^ The authors then present ad hoc proposals to account for long-range interactions in their fourth-generation neural network approach. Namely, they include classical electrostatic interactions that are damped to zero in the short-range. This approach goes back to the origins of small molecule potentials^30^ and more modern versions of it have been incorporated into global methods that do not use range cutoffs, e.g., permutationally invariant polynomial (PIP)-based potentials. Examples include the PIP potentials for CH_5_^+^^31^ and (H_2_O)_2_.^32^ While these do fit data in the long-range, it was also clear that when available, switching to accurate long-range interactions made sense. For example, for the 2-body interaction in a many-body expansion of the water potential q-AQUA, the long-range interaction is switched to the dipole–dipole interaction^33^ using an accurate, flexible dipole moment surface.^34^ In order to avoid “ripples” in the final PES, it is important to verify that the machine-learned (ML) PES overlaps the long-range analytical potential accurately in the region of the switch, as was done in q-AQUA.^33^

In the absence of a high-quality long-range interaction for flexible monomers, we have utilized two component fits.^4^ One component is a precise fit to data in the short-range, and the second one is a fit to data in the long-range. In the example in ref (4) for SiO + H_2_, the long-range data, i.e., CCSD(T) energies, extend to 11.1 Å. The RMS fitting error for the long-range data is 0.05 cm^–1^. This level of precision is just not feasible for a single fit to the entire data set.

In this paper, we present a full-dimensional potential energy surface for the NaCl–H_2_ system, including the correct long-range behavior. The structure of the paper is outlined as follows: Section 2 provides a detailed description of the theoretical and computational aspects, including ab initio calculations, the fitting procedure, long-range interaction, and DMC calculations. The results and properties of the fitted potential energy surface are discussed in Section 3, followed by the summary and conclusions in Section 4.

## 2 Computational Details

**Figure 1:**
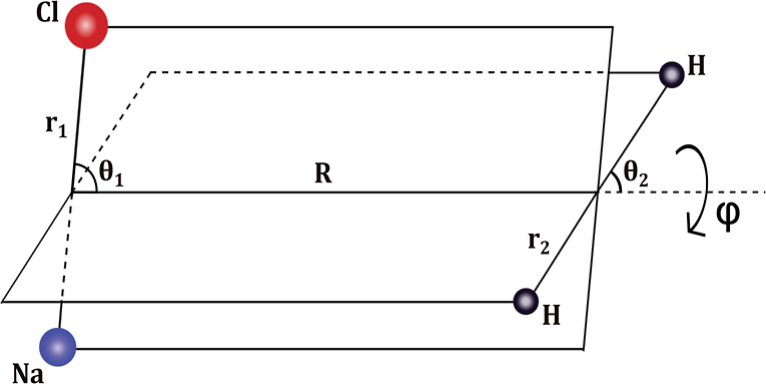
Six-dimensional Jacobi coordinates for the NaCl–H_2_ system.

The interaction potential for the NaCl–H_2_ system in the electronic ground state has been computed using electronic energies generated predominantly on a six dimensional grid defined in Jacobi coordinates (*r*_1_, *r*_2_, *R*, θ_1_, θ_2_, ϕ) as shown in Figure 1. In this context, *R* is the distance between the centers of mass of NaCl and H_2_, while *r*_1_ and *r*_2_ indicate the respective bond lengths of the NaCl and H_2_ molecule. Furthermore, θ_1_ and θ_2_ depict in-plane orientation angles between *r*_1_ and *R*, and *r*_2_ and *R*, while ϕ is the out-of-plane dihedral angle. In the ab initio calculations, *R* is varied in the range of 2.6–12.6 Å, while the bond distances are within the intervals of 2.16 Å ≤ *r*_1_ ≤ 2.66 and 0.50 Å ≤ *r*_2_ ≤ 1.15 Å. The angular coordinates are within the ranges of 0° ≤ θ_1_ ≤ 360° and 0° ≤ θ_2_(ϕ) ≤ 180° where θ_1_ = θ_2_ = 0° corresponds to the collinear arrangement Na–Cl–H–H.

All electronic energy calculations were performed using the coupled-cluster method with single and double excitations and perturbative treatment of triple [CCSD(T)] using the MOLPRO suite of quantum chemistry software programs.^35,36^ The ab initio calculations employed an augmented correlation-consistent polarized valence triple-ζ basis set (aug-cc-pVTZ).^37^

The NaCl–H_2_ potential is given by the “plug and play” form, i.e., it is written as the sum of an interaction potential plus potentials for isolated NaCl and H_2_, as we have done for several potentials.^4,38^ NaCl and H_2_ monomers were fit using the simple sum of powers of the Morse variables. Note that here we employed a straightforward 1-d fit for the NaCl and H_2_ monomers at the CCSD(T)/aug-cc-pVTZ level (abbreviated below as aVTZ). However, users have the flexibility to provide any monomer potential. At each NaCl–H_2_ configuration, the interaction energy is given by the electronic energy of the NaCl–H_2_ minus the electronic energy of NaCl and the electronic energy of H_2_. Clearly, this interaction potential goes to zero asymptotically as NaCl and H_2_ separate. For the previous analogous potential for SiO–H_2_, CCSD(T) energies were obtained for R as large as 11 Å such that the interaction potential is less than 1 cm^–1^.^4^ Note that the long-range interaction for NaCl−H_2_ is stronger than that for SiO + H_2_. To obtain a precise fit to these small asymptotic interaction energies, the PES fit in the short range (*V*_SR_) was joined with the analytical long-range interaction (*V*_LR_) with a smooth switching function as follows
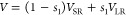
1where
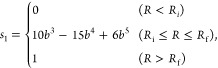
2and *R*_i_ = 10.0 Å, *R*_f_ = 11.0 Å, and *b* = (*R* – *R*_i_)/(*R*_f_ – *R*_i_). Note that the choice of switching range *R*_i_ and *R*_f_ was motivated by the intention to capture a region where the direct CCSD(T) interaction energies exhibit a strong agreement with the analytical dipole–quadrupole interaction.

A large data set of ab initio points was calculated at the CCSD(T) level and used to fit the short-range PES. The PES has been fitted in 6D using an invariant polynomial method.
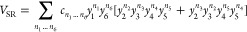
3where  is the linear coefficient and *y*_*i*_ are the Morse variables of form exp(−*d*_*i*_/λ). The parameter λ is subject to user specification, which is set to 3.0*a*_0_ for *V*_SR_. The internuclear distances *d*_*i*_ are denoted as *d*_1_ = *d*_NaCl_, *d*_2_ = *d*_NaH_, *d*_3_ = *d*_NaH_, *d*_4_ = *d*_ClH_, *d*_5_ = *d*_ClH_, and *d*_6_ = *d*_HH_. The total polynomial order for the fits is 7 resulting in a total of 918 linear coefficients. These were obtained using standard linear least-squares, utilizing the MSA software 2.0,^39,40^ which extends the original code^41,42^ to produce and also incorporate gradients data.

### 2.1 Long-Range Interaction between NaCl and H_2_

**Figure 2:**
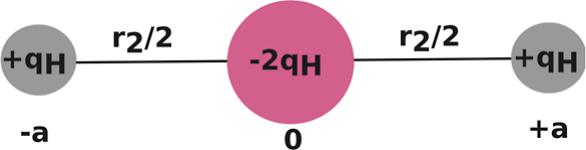
Electric quadrupole moment of the H_2_ molecule.

The leading interaction potential between NaCl and H_2_ in the long-range is given by the dipole–quadrupole interaction^26^
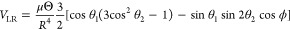
4where μ is the dipole moment of NaCl and Θ is the quadrupole moment of the H_2_ molecule. Instead of using this directly, we use the equivalent expression employing Coulomb’s law
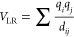
5where *q*_*i*_ are the point charges on NaCl and H_2_ and *d*_*ij*_ are the corresponding distances between the charges on the different molecules. The charges on (*q*_Na_ and *q*_Cl_) are determined using the dipole moment  obtained from ref (43). The charges on the H_2_ molecule are determined by noting that the quadrupole moment can be written as −2*q*_H_ at the center of the molecule as shown in Figure 2. Therefore, the charges of the H_2_ molecule at a given bond length are determined using the quadrupole moment obtained from ref (44). Using the given equation
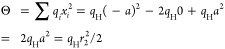
6

**Figure 3:**
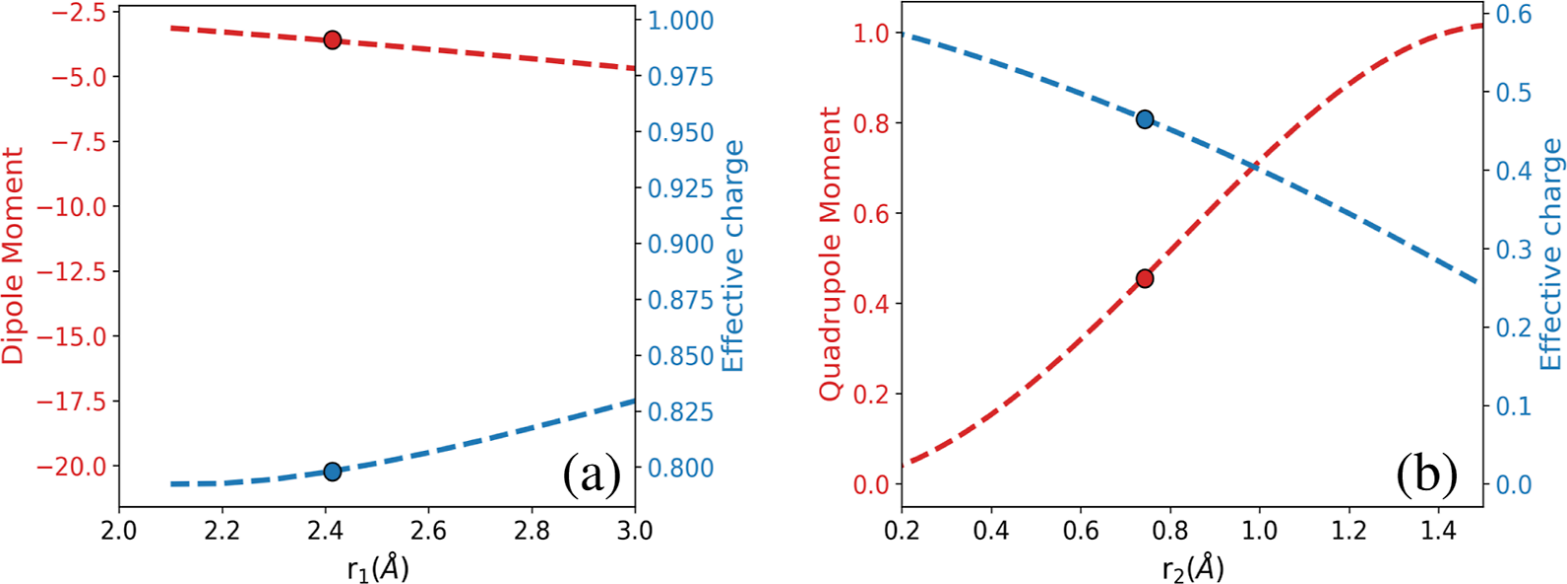
Dipole moment and calculated effective charge as a function of bond length (a) dipole moment and calculated effective charge for NaCl, and (b) quadrupole moment and calculated effective charge for H_2_ molecule, with circles denoting the values at the equilibrium bond lengths. The dipole moment and quadrupole moment data are taken from refs (43) and (44).

Figure 3 shows the variation of the NaCl dipole moment and the corresponding charge and the variation of the H_2_ quadrupole moment and the effective charge with respect to the corresponding internuclear distances. We use eq 5 instead of eq 4 as the former is general and can be used for any term in the multipole expansion. We do note that other shorter range terms, such as the NaCl quadrupole and H_2_ quadrupole interaction, are not considered since the NaCl quadrupole moment as a function of the NaCl distance is not known. Further, as shown below, the leading dipole–quadrupole interaction does match up well to direct CCSD(T) interaction energies.

### 2.2 DMC

Unbiased diffusion Monte Carlo (DMC) calculations^45−47^ were performed to obtain the quantum zero point energy (ZPE) and wave function of the NaCl–H_2_ system using the PES fit and to examine the quality of a PES in extended regions of the configuration space. The fundamental objective of DMC calculation is to solve the time-dependent Schrödinger wave equation in imaginary time τ. DMC calculations begin with an initial guess of the ground-state wave function represented by a population of *N*(0) equally weighted Gaussian random walkers. Subsequently, these walkers diffuse randomly subject to the potential in imaginary time following a Gaussian distribution. Each walker has the possibility to persist (potentially giving birth to a new walker) or be eliminated; the population is regulated by birth-death processes, as described below
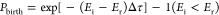
7
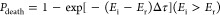
8where *E*_i_ is the energy of the i-th walker, *E*_r_ is the reference energy used to stabilize the diffusion system in its ground state, and Δτ is the step size in imaginary time. To keep the number of random walkers around the initial value *N*(0), *E*_r_ is modified at the end of each time step as per the following equation.
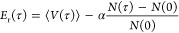
9where *N*(τ) is the number of walkers at the imaginary time step τ, ⟨*V*(τ)⟩ is the average potential energy of all the alive walkers and α is the parameter that regulates the fluctuations in the number of walkers and the reference energy. The average of *E*_r_ over a sufficiently long time period provides an estimate of the ZPE. Here, we performed five DMC calculations, each with an imaginary time step of Δτ = 5 au and α = 0.1. For every DMC calculation, we propagated a set of 20,000 random walkers from the global minimum for 30,000 time steps (≈3.62 ps), out of which 20,000 steps are used to equilibrate the walkers, and the reference energies in the remaining 10,000 steps are used to compute the ZPE. The Cartesian coordinates of the walkers at the last ten steps were recorded, and their interatomic distances were determined.

## 3 Results and Discussion

### 3.1 Precision of the Fits

**Figure 4:**
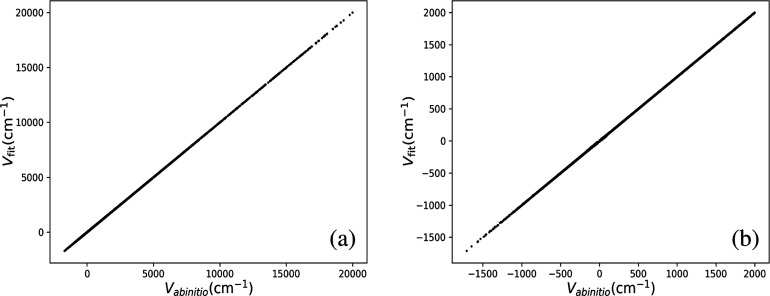
Correlation between fitted and ab initio CCSD(T)/aVTZ interaction energy for the energy cut-off of (a) 20,000, and (b) 2000 cm^–1^.

The potential energy surfaces are fitted using the ab initio data, with a cut-off based on the maximum interaction energy. The precision of the PES fits was determined by calculating the root-mean-square (RMS) fitting errors as a function of the maximum interaction energy. In this work, we choose two interaction energy cut-offs of 20,000 and 2000 cm^–1^ as shown in Table 1. The RMS fitting errors in the short-range fit are 1.42 cm^–1^ for PES I and 1.23 cm^–1^ for PES II. Note that our emphasis is on sampling the ab initio interaction energies essential for achieving an accurate fit. Although opting for an interaction energy range up to 2000 cm^–1^ is a suitable choice, computing additional points in the higher energy range, up to 20,000 cm^–1^, is done for the completeness and to prevent the holes in the PES. Correlation plots are given for the two fits in Figure 4. As seen, there is nearly perfect correlation for both fits over the respective ranges of energies.

**Table 1:** RMS Fitting Error of Two PES Fits as a Function of the Maximum Energy Cut-Off^a^

	*V*_max_	RMS	data points
PES I	20,000	1.42	281,031
PES II	2000	1.23	241,155

a Energies are in cm^–1^.

Table 2 presents the optimized geometrical structure and dissociation energy of the minimum for the PES fits with zero at the relaxed isolated NaCl and H_2_ asymptote, comparing it with the MOLPRO calculated CCSD(T)/aVTZ optimized structures. Hereafter, PES refers to PES I. The global minimum is not for the T-shaped structure, and evidently this indicates that “chemical” effects beyond electrostatics are responsible for the global minimum. It might be interesting for those with more expertise than we have to analyze the source(s) responsible for this structure.

**Table 2:** Optimized Structures of NaCl–H_2_ and Dissociation Energies of *D*_e_ (cm^–1^)^a^

	CCSD(T)/aVTZ	PES I	PES II
*r*_1_	2.4214	2.4214	2.4208
*r*_2_	0.7497	0.7488	0.7487
*R*	2.7931	2.8400	2.8521
θ_1_	113.9	114.6	115.4
θ_2_	42.8	43.7	44.4
ϕ	180.0	180.0	180.0
*D*_e_	632	646	642

a Distances are in Å and angles are in degrees.

**Figure 5:**
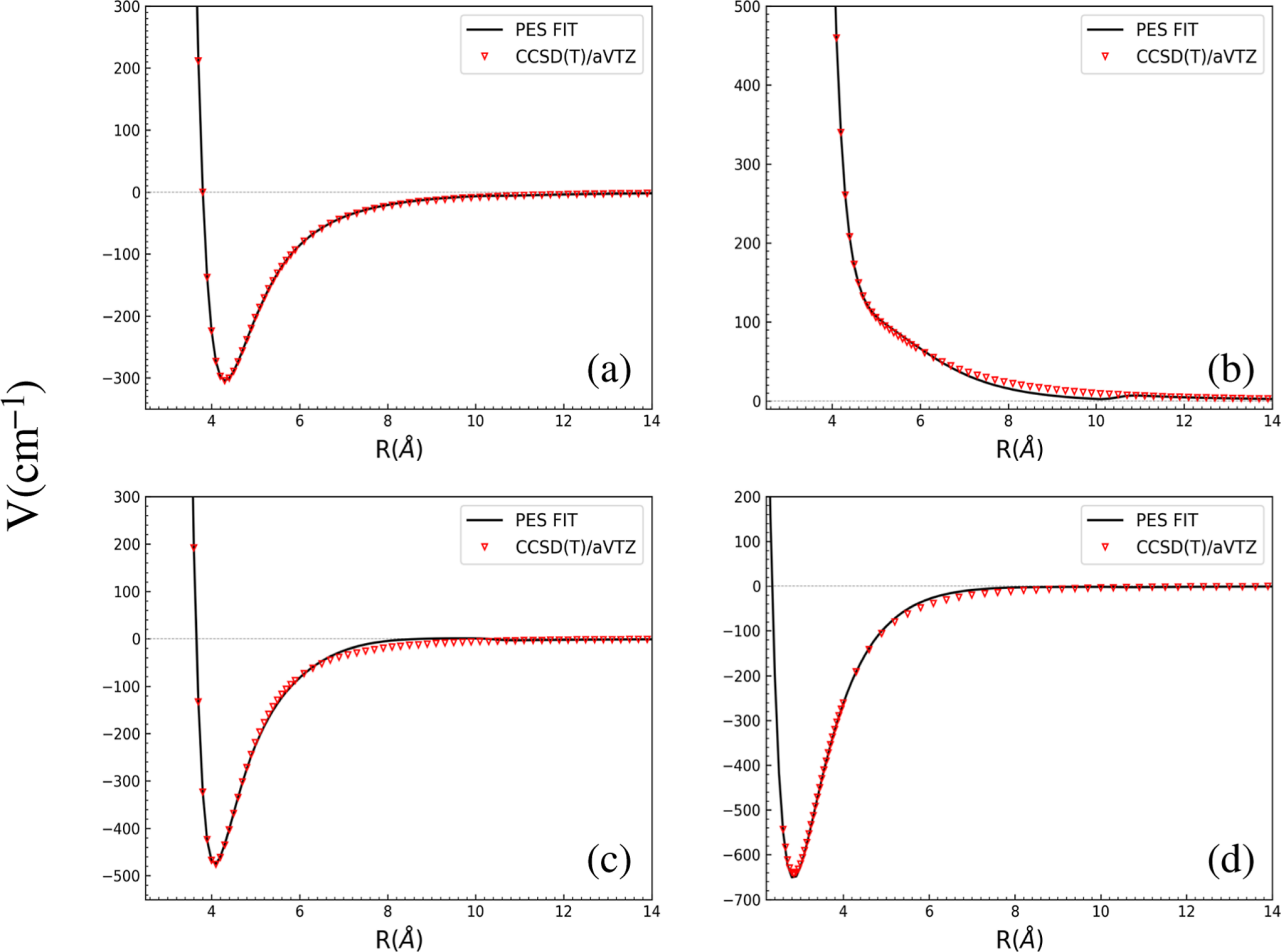
Comparison of several 1D cuts for the interaction energy between NaCl–H_2_ as a function of internuclear distance *R* for various sets of (θ_1_, θ_2_, ϕ): (a) (0, 0, 0), (b) (180, 0, 0), (c) (180, 90, 0), and (d) (114.6, 43.7, 180).

In Figure 5, several 1D cuts for the interaction energy between NaCl–H_2_ are shown at different (θ_1_, θ_2_, ϕ) values from PES and are compared with the ab initio calculations using the aVTZ basis sets. The bond distances of NaCl and H_2_ are fixed at their equilibrium values, *r*_1_ = 2.413 Å and *r*_2_ = 0.743 Å for Figure 5a–c, whereas for Figure 5d, bond lengths are fixed to the values obtained from the optimized minimum geometry listed in Table 2. As seen, over the large range shown, there is excellent agreement between direct CCSD(T) energies and the PES, including in the long-range, *R* greater than 11 Å where the PES is given by the analytical dipole–quadrupole interaction. Note in panel (b) there is a small sudden variation in the PES energy at 10 Å where the switching to the analytical interaction begins. This is actually due to a correction to the PES that slightly underestimates the ab initio energies between around 8–10 Å. The switch restores excellent agreement with the ab initio energies.

**Figure 6:**
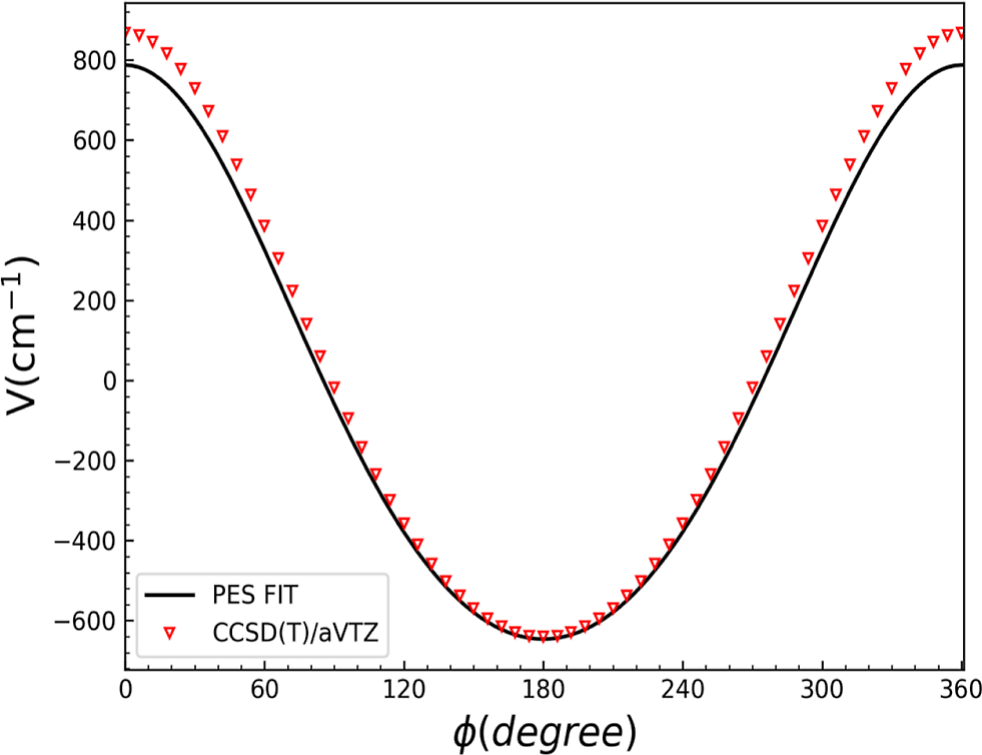
Potential energy cut of NaCl–H_2_ as a function of ϕ.

Figure 6 shows the potential energy cut as a function of ϕ. All other coordinates are fixed in the NaCl–H_2_ equilibrium configuration. As seen, there is a large energy change of roughly 1400 cm^–1^, suggesting that the complex will maintain a mostly planar configuration.

### 3.2 Long-Range Interaction

**Figure 7:**
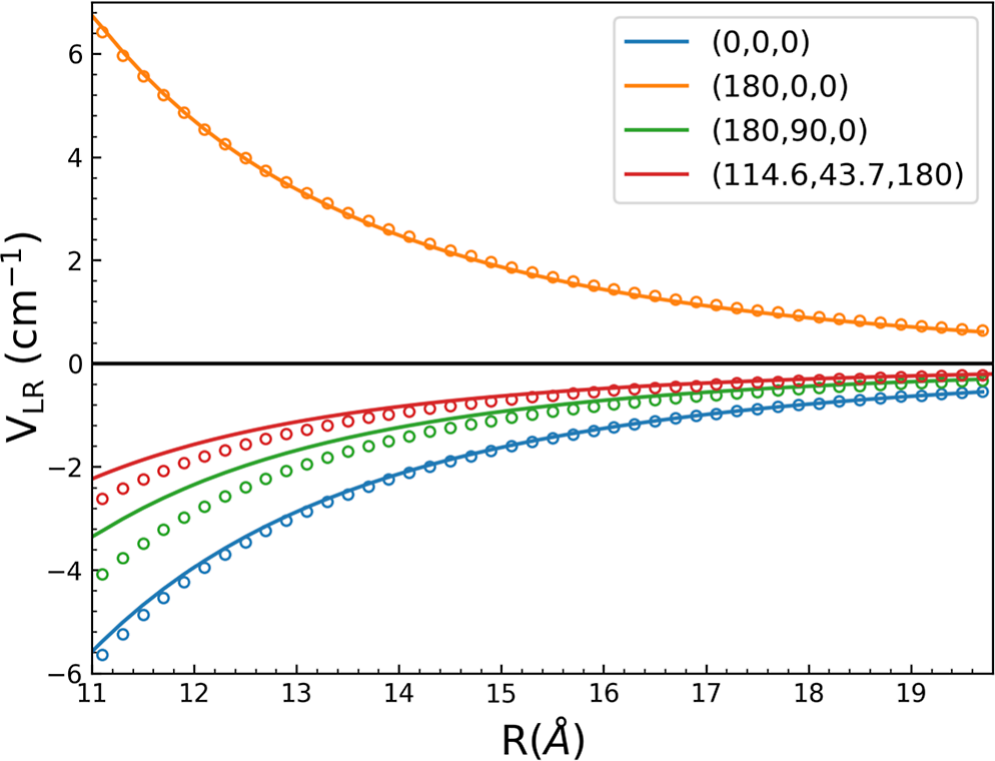
Long-range dipole–quadrupole interaction (solid lines) and corresponding ab initio energies (open circles) vs *R* for indicated (θ_1_, θ_2_, ϕ) sets.

A close examination of the dipole–quadrupole interaction is shown in Figure 7, along with direct CCSD(T) energies. As seen, the maximum deviation of the long-range interaction from the direct CCSD(T) energies is less than 1 cm^–1^ for *R* = 11 Å, which becomes almost exact with a further increase in R.

### 3.3 Zero-Point Wave Function

**Figure 8:**
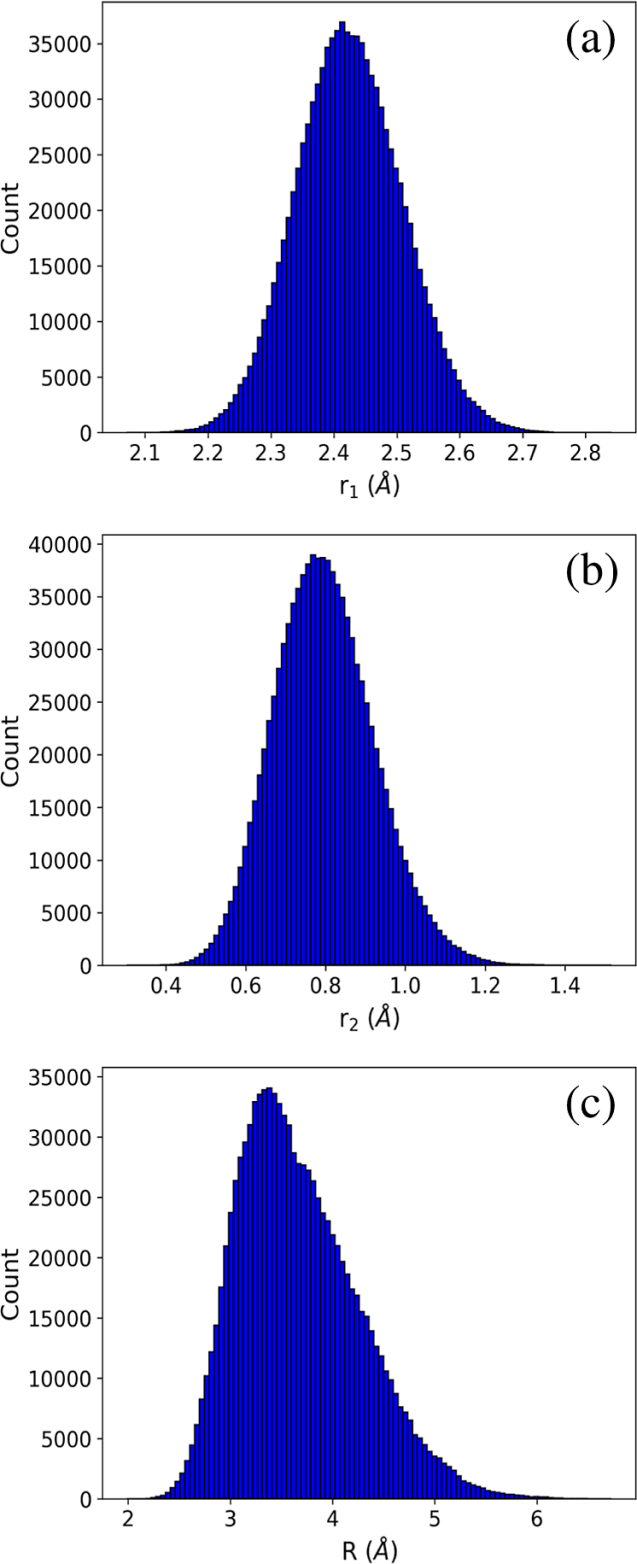
Histogram of cuts of the ground state DMC wave function vs the NaCl and H_2_ internuclear distances, *r*_1_ and *r*_2_, respectively, and *R*.

Next, we present results from the DMC calculations. When we conducted the unconstrained DMC, the PES was found to be “hole-free”, meaning that the PES does not contain any configurations with unphysical negative energies. The average ZPE obtained from five independent DMC simulations using the PES is 2778.16 ± 1.72 cm^–1^. Figure 8 shows histograms of the wave function vs the NaCl and H_2_ internuclear distances, *r*_1_ and *r*_2_, respectively, and *R*. As seen, the results for *r*_1_ and *r*_2_ closely resemble harmonic oscillator wave functions for the ground state. For *R*, where the motion is strongly anharmonic, the wave function displays an asymmetry which has more amplitude at large *R*-values than at small values.

## 4 Summary and Conclusions

We reported a new potential energy surface for NaCl + H_2_ from precise fitting of thousands of CCSD(T)/aug-cc-pVTZ energies. This PES is notable, as it is extended to infinite separation of NaCl and H_2_ by means of an accurate dipole–quadrupole interaction for the flexible monomers. The PES is expressed as the sum of an interaction potential plus flexible monomer potentials, using the “plug and play” strategy adopted previously.^38^

The electronic energies can be accessed at ref (48) and the PES is available as Supporting Information.

## Supporting Information Available

The potential energy surface is supplied as Supporting Information (ZIP)

## References

[ref1] Galli D.; Palla F. The dawn of chemistry. Annu. Rev. Astron. Astrophys. 2013, 51, 163–206. 10.1146/annurev-astro-082812-141029.

[ref2] Roueff E.; Lique F. Molecular excitation in the interstellar medium: Recent advances in collisional, radiative, and chemical processes. Chem. Rev. 2013, 113, 8906–8938. 10.1021/cr400145a. 24131323

[ref3] Zuo J.; Croft J. F.; Yao Q.; Balakrishnan N.; Guo H. Full-Dimensional Potential Energy Surface for Ro-vibrationally Inelastic Scattering between H_2_ Molecules. J. Chem. Theor. Comput. 2021, 17, 6747–6756. 10.1021/acs.jctc.1c00882. 34677959

[ref4] Yang B.; Zhang P.; Qu C.; Wang X.; Stancil P.; Bowman J.; Balakrishnan N.; McLaughlin B.; Forrey R. Full-dimensional quantum dynamics of SiO in collision with H_2_. J. Phys. Chem. A 2018, 122, 1511–1520. 10.1021/acs.jpca.7b09762. 29365271

[ref5] Yang B.; Wang X.; Stancil P.; Bowman J.; Balakrishnan N.; Forrey R. Full-dimensional quantum dynamics of rovibrationally inelastic scattering between CN and H_2_. J. Chem. Phys. 2016, 145, 224307 10.1063/1.4971322. 27984886

[ref6] Yang B.; Balakrishnan N.; Zhang P.; Wang X.; Bowman J.; Forrey R.; Stancil P. Full-dimensional quantum dynamics of CO in collision with H_2_. J. Chem. Phys. 2016, 145, 034308 10.1063/1.4958951. 27448888

[ref7] Yao Q.; Morita M.; Xie C.; Balakrishnan N.; Guo H. Globally accurate full-dimensional potential energy surface for H_2_+ HCl inelastic scattering. J. Phys. Chem. A 2019, 123, 6578–6586. 10.1021/acs.jpca.9b05958. 31268323

[ref8] Bakr B. W.; Smith D. G.; Patkowski K. Highly accurate potential energy surface for the He–H_2_ dimer. J. Chem. Phys. 2013, 139, 144305 10.1063/1.4824299. 24116617

[ref9] Trumbo S. K.; Brown M. E.; Hand K. P. Sodium chloride on the surface of Europa. Sci. Adv. 2019, 5, eaaw7123 10.1126/sciadv.aaw7123. 31206026 PMC6561749

[ref10] Ginsburg A.; McGuire B.; Plambeck R.; Bally J.; Goddi C.; Wright M. Orion SrcI’s disk is salty. Astrophys. J. 2019, 872, 54 10.3847/1538-4357/aafb71.

[ref11] Rivera-Valentín E. G.; Chevrier V. F.; Soto A.; Martínez G. Distribution and habitability of (meta) stable brines on present-day Mars. Nat. Astron. 2020, 4, 756–761. 10.1038/s41550-020-1080-9. 33344776 PMC7745847

[ref12] Acharyya K.; Herbst E. Sodium-Bearing Species in Interstellar Environments. 44th COSPAR Scientific Assembly, 2022; Vol. 44, p 2800.

[ref13] Dai D. J.; Ewing G. E. Induced infrared absorption of H_2_, HD, and D_2_ physisorbed on NaCl films. J. Chem. Phys. 1993, 98, 5050–5058. 10.1063/1.464959.

[ref14] Grunwald M.; Ewing G. E. A two-dimensional quantum crystal: H_2_ on NaCl (100). J. Chem. Phys. 1998, 109, 4990–4996. 10.1063/1.477111.

[ref15] Heidberg J.; Voßberg A.; Hustedt M.; Thomas M.; Briquez S.; Picaud S.; Girardet C. Monolayers of ortho-H_2_, para-H_2_, para-D_2_ and normal-H_2_ adsorbed on NaCl (001) single crystal surfaces. J. Chem. Phys. 1999, 110, 2566–2578. 10.1063/1.477963.

[ref16] Heidberg J.; Gushanskaya N.; Schönekäs O.; Schwarte R. Induced infrared spectra of H_2_ adsorbed on alkali halide surfaces: separation of ortho-and para-H2 by desorption. Surf. Sci. 1995, 331–333, 1473–1478. 10.1016/0039-6028(95)00222-7.

[ref17] Toennies J. P.; Traeger F. The structures and vibrations of H_2_ monolayers on NaCl, MgO and LiF: similarities and differences. J. Phys.: Condens. Matter 2007, 19, 305009 10.1088/0953-8984/19/30/305009.

[ref18] Dawoud J.; Sallabi A.; Jack D. A Monte Carlo simulation study of H2 layers on NaCl (0 0 1). Appl. Surf. Sci. 2008, 254, 7807–7811. 10.1016/j.apsusc.2008.02.083.

[ref19] Zhu Z.; Cao Y.; Zheng Z.; Chen D. An accurate model for estimating H_2_ solubility in pure water and aqueous NaCl solutions. Energies 2022, 15, 5021 10.3390/en15145021.

[ref20] Anstine D. M.; Isayev O. Machine Learning Interatomic Potentials and Long-Range Physics. J. Phys. Chem. A 2023, 127, 2417–2431. 10.1021/acs.jpca.2c06778. 36802360 PMC10041642

[ref21] Martí C.; Laganà A.; Pacifici L.; Pirani F.; Coletti C. A quantum–classical study of the effect of the long range tail of the potential on reactive and inelastic OH+ H_2_ dynamics. Chem. Phys. Lett. 2021, 769, 138404 10.1016/j.cplett.2021.138404.

[ref22] Dörfler A. D.; Eberle P.; Koner D.; Tomza M.; Meuwly M.; Willitsch S. Long-range versus short-range effects in cold molecular ion-neutral collisions. Nat. Commun. 2019, 10, 5429 10.1038/s41467-019-13218-x. 31780657 PMC6882903

[ref23] Grimme S. Density functional theory with London dispersion corrections. Wiley Interdiscip. Rev. Comput. Mol. Sci. 2011, 1, 211–228. 10.1002/wcms.30.

[ref24] Grimme S.; Parac M. Substantial errors from time-dependent density functional theory for the calculation of excited states of large π systems. ChemPhysChem 2003, 4, 292–295. 10.1002/cphc.200390047. 12674603

[ref25] Dreuw A.; Weisman J. L.; Head-Gordon M. Long-range charge-transfer excited states in time-dependent density functional theory require non-local exchange. J. Chem. Phys. 2003, 119, 2943–2946. 10.1063/1.1590951.

[ref26] Stone A. The theory of intermolecular forces; OUP oxford, 2013.

[ref27] Zhai Y.; Caruso A.; Bore S. L.; Luo Z.; Paesani F. A “short blanket” dilemma for a state-of-the-art neural network potential for water: Reproducing experimental properties or the physics of the underlying many-body interactions?. J. Chem. Phys. 2023, 158, 084111 10.1063/5.0142843. 36859071

[ref28] Xia J.; Zhang Y.; Jiang B. Accuracy Assessment of Atomistic Neural Network Potentials: The Impact of Cutoff Radius and Message Passing. J. Phys. Chem. A 2023, 127, 9874–9883. 10.1021/acs.jpca.3c06024. 37943102

[ref29] Ko T. W.; Finkler J. A.; Goedecker S.; Behler J. A fourth-generation high-dimensional neural network potential with accurate electrostatics including non-local charge transfer. Nat. Commun. 2021, 12, 398 10.1038/s41467-020-20427-2. 33452239 PMC7811002

[ref30] Tang K. T.; Toennies J. P. An improved simple model for the van der Waals potential based on universal damping functions for the dispersion coefficients. J. Chem. Phys. 1984, 80, 3726–3741. 10.1063/1.447150.

[ref31] Qu C.; Yu Q.; Houston P. L.; Pandey P.; Conte R.; Nandi A.; Bowman J. M. Diffusion Monte Carlo and PIMD calculations of radial distribution functions using an updated CCSD (T) potential for CH_5_^+^. Mol. Phys. 2023, e2262058 10.1080/00268976.2023.2262058.

[ref32] Nandi A.; Qu C.; Houston P. L.; Conte R.; Yu Q.; Bowman J. M. A CCSD (T)-based 4-body potential for water. J. Phys. Chem. Lett. 2021, 12, 10318–10324. 10.1021/acs.jpclett.1c03152. 34662138

[ref33] Yu Q.; Qu C.; Houston P. L.; Conte R.; Nandi A.; Bowman J. M. q-AQUA: A Many-Body CCSD(T) Water Potential, Including Four-Body Interactions, Demonstrates the Quantum Nature of Water from Clusters to the Liquid Phase. J. Phys. Chem. Lett. 2022, 13, 5068–5074. 10.1021/acs.jpclett.2c00966. 35652912

[ref34] Lodi L.; Tennyson J.; Polyansky O. L. A Global, High Accuracy Ab Initio Dipole Moment Surface for the Electronic Ground State of the Water Molecule. J. Chem. Phys. 2011, 135, 034113 10.1063/1.3604934. 21786993

[ref35] Werner H. J.; Knowles P. J.; Knizia G.; Manby F. R.; Schütz M. Molpro: a general-purpose quantum chemistry program package. Wiley Interdiscip. Rev. Comput. Mol. Sci. 2012, 2, 242–253. 10.1002/wcms.82.

[ref36] Werner H. J.; Knowles P. J.; Manby F. R.; Black J. A.; Doll K.; Heßelmann A.; Kats D.; Köhn A.; Korona T.; Kreplin D. A.; et al. The Molpro quantum chemistry package. J. Chem. Phys. 2020, 152, 144107 10.1063/5.0005081. 32295355

[ref37] Kendall R. A.; Dunning T. H. Jr.; Harrison R. J. Electron affinities of the first-row atoms revisited. Systematic basis sets and wave functions. J. Chem. Phys. 1992, 96, 6796–6806. 10.1063/1.462569.

[ref38] Qu C.; Conte R.; Houston P. L.; Bowman J. M. Plug and play full-dimensional ab initio potential energy and dipole moment surfaces and anharmonic vibrational analysis for CH_4_–H_2_O. Phys. Chem. Chem. Phys. 2015, 17, 8172–8181. 10.1039/C4CP05913A. 25726765

[ref39] MSA Software with Gradients, 2019. https://github.com/szquchen/MSA-2.0, accessed Jan 1 20, 2019.

[ref40] Houston P. L.; Qu C.; Yu Q.; Conte R.; Nandi A.; Li J. K.; Bowman J. M. PESPIP: Software to fit complex molecular and many-body potential energy surfaces with permutationally invariant polynomials. J. Chem. Phys. 2023, 158, 044109 10.1063/5.0134442. 36725524

[ref41] Xie Z.; Bowman J. M. Permutationally invariant polynomial basis for molecular energy surface fitting via monomial symmetrization. J. Chem. Theor. Comput. 2010, 6, 26–34. 10.1021/ct9004917. 26614316

[ref42] MSA Software 1.0 with Video. 2016, https://scholarblogs.emory.edu/bowman/msa/.

[ref43] Wang Y.; Bowman J. M.; Kamarchik E. Five ab initio potential energy and dipole moment surfaces for hydrated NaCl and NaF. I. Two-body interactions. J. Chem. Phys. 2016, 144, 114311 10.1063/1.4943580. 27004880

[ref44] Wolniewicz L.; Simbotin I.; Dalgarno A. Quadrupole transition probabilities for the excited rovibrational states of H_2_. Astrophys. J. Suppl. 1998, 115, 293–313. 10.1086/313091.

[ref45] Anderson J. B. A random-walk simulation of the Schrödinger equation: H_3_^+^. J. Chem. Phys. 1975, 63, 1499–1503. 10.1063/1.431514.

[ref46] Kosztin I.; Faber B.; Schulten K. Introduction to the diffusion Monte Carlo method. Am. J. Phys. 1996, 64, 633–644. 10.1119/1.18168.

[ref47] McCoy A. B. Diffusion Monte Carlo Approaches for Investigating the Structure and Vibrational Spectra of Fluxional Systems. Int. Rev. Phys. Chem. 2006, 25, 77–107. 10.1080/01442350600679347.

[ref48] Electronic interaction energies of NaCl+H2. 2023, https://github.com/jmbowma/QM-22, accessed Dec 20, 2023.

